# Impact of temperature and soil type on *Mycobacterium bovis* survival in the environment

**DOI:** 10.1371/journal.pone.0176315

**Published:** 2017-04-27

**Authors:** Elodie Barbier, Murielle Rochelet, Laurent Gal, Maria Laura Boschiroli, Alain Hartmann

**Affiliations:** 1 Agroécologie, AgroSup Dijon, INRA, Université de Bourgogne Franche Comté, Dijon Cedex, France; 2 Université Paris-Est, Laboratoire National de Référence de la Tuberculose, Unité de Zoonoses Bactériennes, Laboratoire de Santé Animale, ANSES, Maisons-Alfort Cedex, France; University of Padova, Medical School, ITALY

## Abstract

*Mycobacterium bovis*, the causative agent of the bovine tuberculosis (bTB), mainly affects cattle, its natural reservoir, but also a wide range of domestic and wild mammals. Besides direct transmission *via* contaminated aerosols, indirect transmission of the *M*. *bovis* between wildlife and livestock might occur by inhalation or ingestion of environmental substrates contaminated through infected animal shedding. We monitored the survival of *M*. *bovis* in two soil samples chosen for their contrasted physical and-chemical properties (i.e. pH, clay content). The population of *M*. *bovis* spiked in sterile soils was enumerated by a culture-based method after 14, 30, 60, 90, 120 and 150 days of incubation at 4°C and 22°C. A qPCR based assay targeting the IS*1561*’ locus was also performed to monitor *M*. *bovis* in both sterile and biotic spiked soils. The analysis of survival profiles using culture-based method showed that *M*. *bovis* survived longer at lower temperature (4°C versus 22°C) whereas the impact of soil characteristics on *M*. *bovis* persistence was not obvious. Furthermore, qPCR-based assay detected *M*. *bovis* for a longer period of time than the culture based method with higher gene copy numbers observed in sterile soils than in biotic ones. Impact of soil type on *M*. *bovis* persistence need to be deepened in order to fill the gap of knowledge concerning indirect transmission of the disease.

## Introduction

*Mycobacterium bovis* is a pathogenic mycobacteria responsible for bovine tuberculosis (bTB). Tuberculosis caused by *M*. *bovis* is a primarily respiratory disease that also affects various organs in animals [1]. Even if bTB mainly concerns cattle, a wide range of domestic and wild mammals can be infected as well [2]. On the other hand, bTB a zoonotic disease impacting human health [3]. Although cattle is considered to be the principal host of *M*. *bovis*, wild fauna such as the badger (*Meles meles*), wild boar (*Sus scrofa*), red deer (*Cervus elaphus*) and brushtail possum (*Trichosurus Vulpecula*) have been identified as potential wildlife reservoirs [4]. Though transmission of *M*. *bovis* among cattle usually occurs *via* inhalation of contaminated aerosols released by infected animals during close contacts between animals [1, 5], indirect transmission either by inhalation of environmental bioaerosols or ingestion of infected environmental matrices may be alternative potential routes of animal infection [5–7]. Indirect transmission through feed or water has already been demonstrated between deers, deers and cattle [7, 8] and badgers and cattle [9] in laboratory experiments.

Investigating this type of indirect transmission is challenging because it results at least from the combination of three essential factors *i*.*e*., i) the environmental contamination by shedding from infected animals, ii) the persistence of the bacteria under a viable state in environmental matrices and finally iii) the interaction between a new susceptible host with the contaminated matrices. The shedding of *M*. *bovis* has already been demonstrated in many species *via* oro-nasal mucus, sputum, urine, feces and wound discharges, depending on the species [7, 10, 11]. Previous experimental studies using various environmental substrates showed that *M*. *bovis* was able to survive for a long-time period outside of its host [12–16]. For example, using a culture-based method, Ghodbane *et al*. [16] recovered viable *M*. *bovis* from sterile soils incubated in controlled laboratory conditions 12 months after their inoculation whereas an incubation under natural weather conditions of Michigan, USA, allowed the persistence of bacteria in sterile soils for 88 days [15]. Moreover, numerous studies have identified several climatic factors such as a low temperature, an adequate moisture and a protection against solar radiation (ultra violet) as positive factors for the persistence of *M*. *bovis* in environmental matrices like feces [12, 17], food supply such as corn, hay, apples [15, 18], water [9, 15] and soil [13, 15, 17, 19]. When Fine *et al*. [15] compared the persistence of *M*. *bovis* in four substrates (corn, hay, water and soil), the longest survival was found in cool and moist soil (88 days in winter/spring) suggesting that this substrate probably ensured better conditions of persistence for *M*. *bovis*. Until now, environmental detection of *M*. *bovis* has been mainly achieved in soil, especially in badger sett soil or in pasture soil [14, 20–23].

To the best of our knowledge, no accurate data on the influence of soil characteristics on *M*. *bovis* persistence are available. A low pH and a high iron content are often described as major factors driving mycobacterial survival in soils, especially for *Mycobacterium avium* ssp. p*aratuberculosis* [24, 25]. Concerning *M*. *bovis*, one study based on a large enquiry in cattle farms established a relationship between the occurrence of bTB in animals and soil parameters thus showing that a 1% increase in the sandy content of soil led to an increase in odds of bTB infection by 4% [26], suggesting that soil type may promote or hinder *M*. *bovis* environmental persistence.

Culture-based method is the gold standard for direct diagnosis of *M*. *bovis* in animals. However this method is unsuitable for analysing environmental samples due to the abundance and diversity of soil microorganisms and to the slow growth rate of *M*. *bovis*. Moreover, pre-treatments applied to prevent competition of fast growing organisms, such as a harsh decontamination, strongly reduce *M*. *bovis* viability [14, 15]. That is why molecular detection tools such as semi-quantitative PCR or real-time PCR (qPCR) have been preferentially used to detect *M*. *bovis* environmental samples [14, 20, 21, 23].

The objective of this study was to further assess the role of the soil characteristics on persistence of *M*.*bovis*. Thus, the effects of both temperature and physicochemical characteristics of the soil on the survival of *M*. *bovis* were studied under controlled laboratory conditions. For this purpose, two different soils selected for their contrasted properties either sterile or natural (biotic) were spiked with known concentrations of *M*. *bovis* and incubated at two different temperatures to mimic seasonal temperature variation. The persistence of *M*. *bovis* over the time was monitored using a culture-based method for the detection of viable *M*. *bovis* and a qPCR-based assay.

## Materials and methods

### *Mycobacterium bovis* cultivation and inoculum preparation

The strain *Mycobacterium bovis* “Côte d’Or” SB0120 VNTR 5 5 4 3 11 4 5 6 that infects cattle and wild animals such as badgers and wild boar in France and particularly in Côte d’Or [27] was provided by the National Reference Laboratory of bovine tuberculosis (Maisons-Alfort, France). The strain was grown aerobically at 37°C in Middlebrook 7H9 supplemented with 10% Middlebrook ADC Growth Supplement (Sigma-Aldrich, France) for thirty days. The *M*. *bovis* inoculum was prepared as following: a 20 mL stationary phase culture was pelleted and once the supernatant removed the cell aggregates were dissociated by vortexing with glass beads for 15 seconds. The pellet was suspended in 1 mL of sterile water constituting the stock solution and serial 10-fold dilutions were subsequently plated in triplicate onto Middlebrook 7H11 supplemented with 10% heat inactivated bovine serum (Dominique Dutscher, France) and 10% Middlebrook OADC Growth Supplement (Sigma-Aldrich, France) for enumeration. Colonies were counted following a two-months incubation time at 37°C. The inoculum for the culture-based assay and the qPCR-based assay were respectively prepared by diluting 10-fold and 1000-fold the *M*. *bovis* stock solution. Unless otherwise stated, all experiments were done in a biosafety level 3 laboratory.

### Soil characterization and microcosm preparation

Two different soils were collected in two cattle-grazed pastures at 0–20 cm depth in Côte d’Or region (middle east, France). Soil A (latitude: 47,245201°E, longitude: 4,648302°N) is a clay loamy soil (calcisol) and soil B (latitude: 47,396577°E, longitude: 4,230354°N) is a loamy sandy soil. Soils were collected from privately owned fields, once the owners agreed with soil sampling and subsequent use of the soils for experimental purposes under laboratory conditions.

Soils were sieved to 4 mm and stored at 4°C until used (up to one month). Physical and chemical characteristics of each soil were determined by the Soil Analysis Laboratory of Arras, (LAS, INRA, Arras, France).

Microcosms were prepared by filling conical vials of 15 mL (Falcon, Dominique Dutscher, France) with 2 g of soils A or B. For each soil, two types of microcosms were set up: half of the vials were prepared using fresh soil(stored at 4°C) and the other half using sterilized soil. Sterilized soil was obtained from air dried soil, by gamma-irradiation up to an internal minimum dose of 35 KGray, (Ionisos, France). In order to ensure that soil moisture would not interfere with *M*. *bovis* survival, the soil microcosms (biotic and sterilized) were then adjusted to 90% of the water holding capacity (WHC) using sterile deionized water. We checked that neither soil A nor B were positive for *M*. *bovis* with the culture-based method (sterile soils) and with the qPCR assay (sterile and biotic soils) described later. All the soil microcosms were spiked with 100 μL of the 10- or 1000-fold diluted *M*. *bovis* inoculum and stored in the dark in a biosafety level 3 laboratory until their analysis. Sterile spiked soils were incubated at 4°C and 22°C while biotic spiked soils were only incubated at 22°C. Vials were opened under the safety cabinet every fifteen days to renew the vial atmosphere.

### DNA extraction and purification

DNAs were extracted from the soil samples as follows: 8 mL of lysis buffer (100 mM Tris pH 8.0, 100 mM EDTA pH 8.0, 100 mM NaCl and 2% (w/v) SDS), 4 g of silica beads (100 μm diameter), 5 g of ceramic beads (1.4 mm diameter) and 8 glass beads (4 mm diameter) were added to each vial of soil. However, as DNA extraction could not be achieved under biosafety level 3 laboratory for practical reasons, soil was suspended in 8 mL of lysis buffer and heated 2 hours at 80°C to kill viable bacteria. The following steps of DNA extraction were then done in a biosafety level 2 laboratory. Samples were disrupted for 3 × 30 s at 4 m/s in a FastPrep®-24 Instrument (MP Biomedicals Europe, France), incubated for 30 min at 70°C and centrifuged for 5 min at 7000 ×*g* at room temperature. Supernatants were incubated on ice for 10 min after adding 1/10 of their volume of 3 M potassium acetate pH 5.5 and then centrifuged for 5 min at 14000 × *g*. One volume of ice-cold isopropanol was added to the supernatant for DNA precipitation overnight at − 20°C. DNA was collected by centrifugation (30 min at 14000 × *g*), and DNA pellets were washed with ice-cold ethanol (70%) and dissolved in 100 μL of water. Crude DNA extracts (100 μL) were loaded on Microbiospin™ columns (Biorad, France) filled with 100 mg of polyvinyl polypyrrolidone (PVPP, Sigma Chemical Co, France). After centrifugation (4 min at 1000 × *g*, 4°C), eluted DNA was further purified using the Geneclean Turbo Kit (Qiagen, France) according to the manufacturer's instructions. Purified DNA concentrations were estimated by spectrophotometry (λ = 260 nm) using a NanoDrop® 2000 spectrophotometer (Thermo Scientific, France).

### Estimation of DNA quality for PCR amplification

The quality of the purified DNAs extracted from the two soils was assessed by measuring the capacity to amplify a control DNA target in the presence of various dilutions of soil DNA extracts. Briefly, qPCR reactions were done in a final volume of 25 μL containing 2.5 μL of 10^6^ copies of the circularized pGEM-T Easy plasmid DNA and 5 μL of water (control) or diluted soil DNA (10- and 20-fold dilutions), 1X Absolute qPCR SYBR Green ROX (500 nM) Mix (Thermo Scientific), 25ng/μL of T4 bacteriophage gene 32 product. qPCR primers were SP6 (5’–ATTTAGGTGACACTATAG-3’) and T7 (5’-TAATACGACTCACTATAGGG-3’) universal primers (final concentration 500 nM) targeting the polylinker of the plasmid. qPCR and cycling conditions were as following: initial denaturation at 95°C for 15 min, followed by 40 cycles with denaturation at 95°C for 15 s, annealing at 55°C and elongation at 72°C for 30 s each. The PCR product specificity was assessed by the melting curve analysis. Reduction of the pGEM-T apparent copy numbers in the presence of diluted soil DNA compared to the copy number obtained in the control reaction (water without soil DNA) was used to estimate qPCR inhibition caused by impurities contained in soil DNA samples.

### Cuture-based method

The sterile soil microcosms (m = 2 g) were suspended in 9 mL of Middlebrook 7H9 supplemented with ADC by vortexing the vials for 2 min. Tenfold dilutions of the soil suspensions were achieved and 100 μL were plated in triplicate onto Middlebrook 7H11 agar enriched as described above and supplemented with antibiotics and antifungal (100 mg.L^-1^ Ticarcillin, 10 mg.L^-1^ Trimethoprim, 200 000 UI.L^-1^ Polymyxin B and 100 mg.L^-1^ Cycloheximid, Sigma-Aldrich, France) for enumeration. Plates were then incubated in gas-permeable bags at 37°C for 2 months and checked every 15 days.

### *Mycobacterium tuberculosis* complex quantitative PCR (qPCR)

*Mycobacterium bovis* was detected using the *Mycobacterium tuberculosis* complex (MTBC) specific qPCR targeting the IS*1561’* locus (Forward: 5’- GATCCAGGCCGAGAGAATCTG -3’, Reverse: 5’- GGACAAAAGCTTCGCCAAAA—3’ and probe 5’—FAM ACGGCGTTGATCCGATTCCGC TAMRA—3’) as previously described [28].

qPCR reactions were carried out in triplicate with a 25 μL reaction mix containing 12.5 μL of ABsolute™ QPCR ROX Mix (Thermo Scientific, France), 1 μL (final concentration 25 ng.μL ^- 1^) of T4 bacteriophage gene 32 product (MP Biomedicals Europe, France), 1 μL of each primer (final concentration 400 nmol.L ^-1^), 0.5 μL of each probe (final concentration 200 nmol. L ^-1^), 4 μL of ultrapure water and 5 μL of 20-fold diluted DNA. Absolute quantification was achieved using standard DNA dilutions. A recombinant plasmid containing one copy of the IS*1561*’ fragment of *M*. *bovis* BCG strain Pasteur 1173P2 cloned in the pCR^®^II-TOPO^®^ vector (Invitrogen, France) was used as a standard. A calibration curve was obtained by amplification of serial dilutions of the plasmid ranging from 1 to 10^6^ copies per PCR reaction. All DNAs extracted from the soil samples were screened with the IS*1561*’-based qPCR assay and results were expressed as the number of *M*. *bovis* gene copy number per g of soil. qPCR assay was performed in a VIIA 7 Real-Time PCR System (Thermo Fischer Scientific, France). Initial denaturation was done at 95°C for 15 min, followed by fourty-five cycles with denaturation at 95°C for 15 s, annealing and elongation at 60°C for 1 min. Detection limits of this molecular system was previously determined in soil [28].

### Schedule of microcosm sampling

In order to monitor *M*. *bovis* cell numbers over time, both plate enumeration and qPCR assay were performed on irradiated soil microcosms, while only qPCR assay was carried out for biotic soil microcosms. At each sampling date *i*.*e*. day 0, 14, 30, 60, 90, 120 and 150, three microcosms (3 independent replicates) of each biotic and sterile soils were analysed with the qPCR assay after DNA extraction/purification and four microcosms (4 independent replicates) of each sterile soil were analysed with culture-based method.

## Results

### Physico-chemical characteristics of soils A and B

The physico-chemical characteristics of soils A and B are summarized in Table 1. The soil A was a clay loamy soil with a slightly basic pH (7.75) and a high content of clay (clay account for 39.7% in particle-size distribution and sand for only 11.8%) while the soil B was a loamy sandy soil with an acidic pH (5.46) and a high content of sand (sand account for 34.5% in particle-size distribution and clay for only 19.2%). Silt content was roughly equivalent in the two soils (485 g kg^-1^ for soil A *vs* 463 for soil B). We chose to adjust soil moisture to 90% of their water field holding capacity leading to a moisture content of 31% for soil A and 21% for soil B. Finally, these two soils possessed low levels of nitrogen compounds: the C-to-N ratio was 9.5 for soil A and 10.9 for soil B.

**Table 1:** Physical and chemical parameters of soils A and B.

**Physical parameters**	**Soil A**	**Soil B**	**Unit**
Clay (< 2 μm)	397	192	g kg^-1^
Silt	485	463	g kg^-1^
Sand	118	345	g kg^-1^
Water field capacity	352	247	g kg^-1^
**Chemical parameters**	Soil A	Soil B	Unit
pH	7.75	5.46	-
Total C	36.1	29.2	g kg^-1^
Total N	3.8	2.69	g kg^-1^
C- to N- ratio	9.5	10.9	-
Organic matter	62.5	50.6	g kg^-1^
P	0.031	0.025	g kg^-1^
K	0.158	0.0877	g kg^-1^
Ca	7.17	1.1	g kg^-1^
Na	0.0115	0.0129	g kg^-1^
Mg	0.0541	0.0789	g kg^-1^
Fe	0.526	0.227	g kg^-1^
CEC^a^	18.3	10.8	cmol+.kg^-1^ ^b^

^a^CEC: Cation Exchange Capacity

^b^cmol+.kg^-1^: centimoles of positive cations per kg of soil

### Effect of temperature and soil type on *M*. *bovis* survival time measured by culture-based method

**Fig 1:**
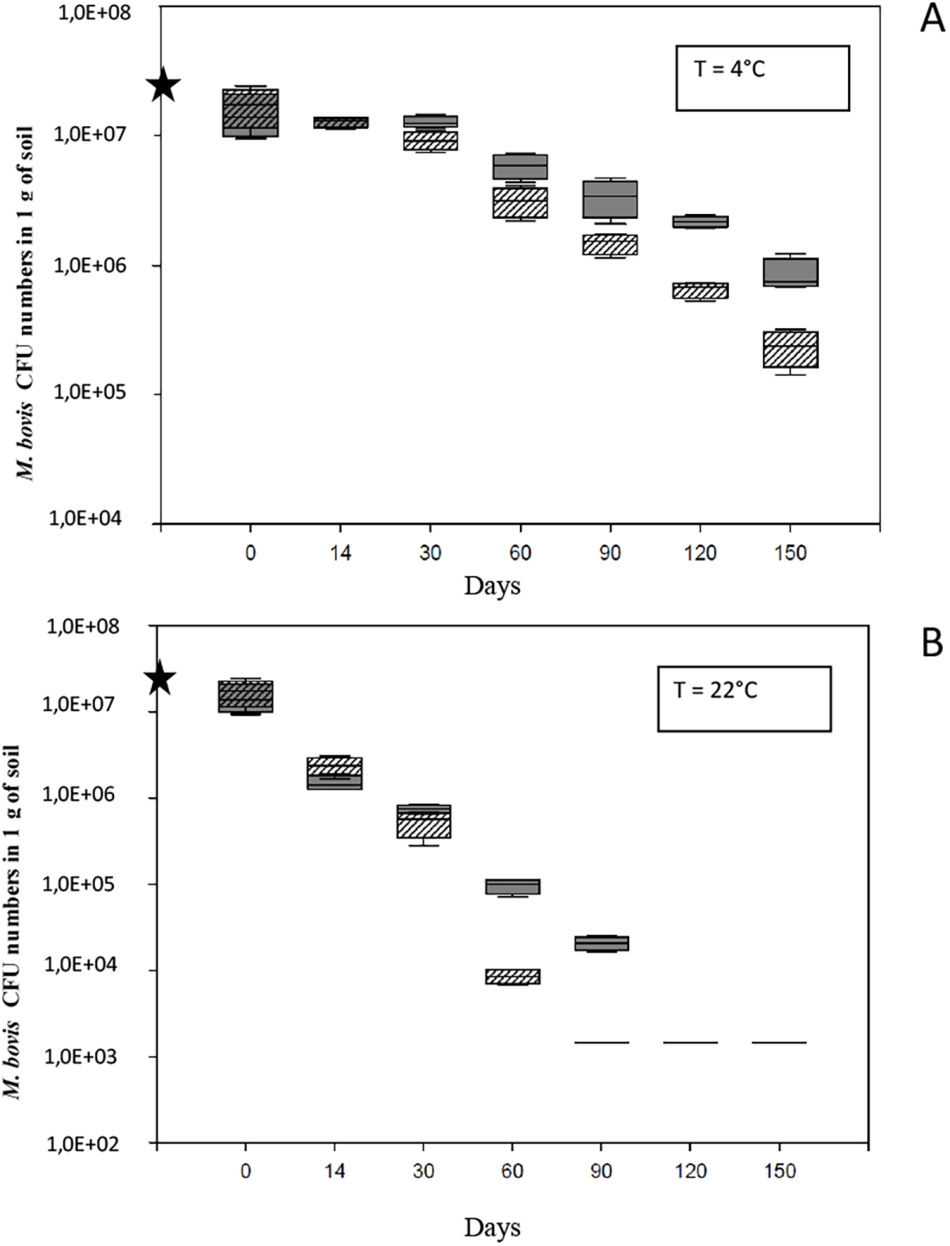
Box-plot graph showing the survival of *M*. *bovis* SB0120 along the time by a culture-based method in sterile soils: soil A (solid gray symbols) and soil B (symbols with gray stripes) after incubation at 4°C (Panel A) and at 22°C (Panel B). The black star symbol on the y-axis represents the inoculation level of *M*. *bovis* BCG in soil microcosms. Error bars represent the standard deviation values on 4 replicates.

Theoretical detection limit of our culture-based method was estimated at 1.5 × 10^3^ CFU g^-1^ soil. Total cultivable *M*. *bovis* (CFU g^-1^ soil) were enumerated by the culture-based method described above for each sterile soil incubated at 4°C and 22°C at day 0, 14, 30, 60, 90, 120 and 150. At day 0, the numbers of *M*. *bovis* were determined by the culture-based method and values of (1.5 ± 0.6) × 10^7^ CFU g^-1^ and (1.7 ± 0.7) × 10^7^ CFU g^-1^ were estimated for soils A and B (n = 4), respectively. Two different patterns of mycobacterial survival were observed depending on the temperature, (Fig 1). At 22°C, *M*. *bovis* cells decreased over the course of the experiment in the two sterile soils A and B (Fig 1, panel B). No more cultivable bacteria growth was observed from day 90 in soil B and from day 120 in soil A to the end of the experiment at day 150. Although *M*. *bovis* “Côte d’Or” was detected by culture for one month longer in soil A than in soil B, no statistically significant difference of CFU numbers was observed between the two soils at 22°C (Test Student-Newman-Keuls, p = 0.559). Thus, no significant difference in *M*. *bovis* survival was observed for the two different soil types.

Concerning the incubation at 4°C, *M*. *bovis* cultivable cells were recovered with higher concentrations than those observed at 22°C, in the two soils at every sampling date (over the five months of the experiment). As indicated in Fig 1(panel A), the *M*. *bovis* CFU numbers remained stable during the first 30 days in the two soils at 4°C. Then, a slight decrease of the CFU numbers in the two soils starting from day 60 to the end of the study was observed. In both soils, *M*. *bovis* CFU numbers decreased significantly from day 0 to day 150 (Test Student-Newman-Keuls, p<0,001), reaching (8.7 ± 2.5) × 10 ^5^ CFU g^- 1^ and (2.2 ± 1.1) × 10 ^5^ CFU g^- 1^ in soil A and B, respectively.

### Effect of soil type and soil endogenous microbiota on *M*. *bovis* survival time measured by qPCR assay

**Fig 2:**
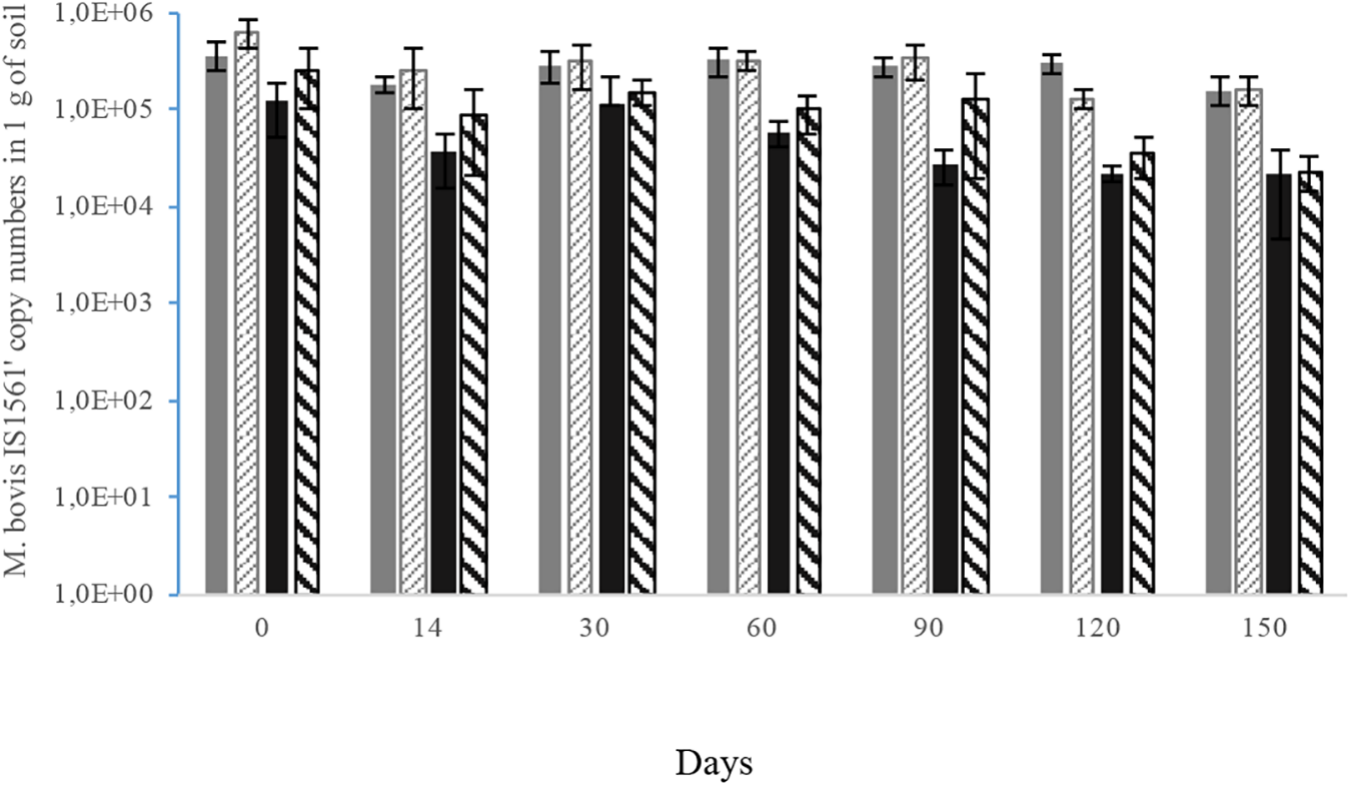
Histogram showing the detection of M. bovis gene copy numbers over the time by the IS1561’-based qPCR assay on sterile soil A (solid grey bars), sterile soil B (gray stripe bars) and biotic soil A (solid black bars), biotic soil B (black stripe bars) after incubation at 22°C. Error bars represent standard deviation values on 3 replicates.

Survival of *M*. *bovis* when biotic or sterile soils A and B were incubated at 22°C was monitored using the qPCR based assay. None of the extracted soil DNA’s induced qPCR inhibition. The *M*. *bovis* gene copy numbers were estimated with IS*1561*’qPCR-based method for each sterile and biotic soils incubated at 22°C at day 0, 14, 30, 60, 90, 120 and 150 (see Fig 2). At day 0, the number of *M*. *bovis* gene copies per g of soil yielded (3.6 ± 1.2) × 10^5^ and (6.0 ± 1.9) × 10^5^ respectively for sterile soils A and B and (1.2 ± 0.7) × 10^5^ and (2.5 ± 1.5) × 10^5^ respectively for biotic soils A and B.

Overall, high amounts of *M*. *bovis* gene copies (ranging from 10^4^ to nearly 10^6^ g^-1^ of soil) were detected in the four soils at the eight sampling dates.

A statistically significant interaction between time and soil condition (biotic *vs* sterile) was observed when considering the two soils independently and dependently (Test Student-Newman-Keuls, p<0,001) with higher amounts of gene copies recovered in sterile soils than in biotic ones from day 0 to day 150. No significant differences were observed when comparing the gene copy numbers in the two sterile soils or in the two biotic soils at each sampling date.

Even though the *M*. *bovis* gene copy numbers slightly oscillated in the four soils over the course of the experiment, a slight but significant decrease of these numbers was observed between day 0 and day 150 for each soil. The highest decrease of *M*. *bovis* numbers were observed in the biotic soils with a rough loss of 1 log for soil B and 0.75 log for soil A between day 0 and day 150 (Fig 2).

Finally, qPCR assay allowed *M*. *bovis* detection in sterile soils A and B incubated at 22°C over a longer period of time than the culture-based assay. Indeed, *M*. *bovis* DNA was detected by qPCR for up to 150 days in the two sterile soil samples, while bacterial culture detected viable *M*. *bovis* cells up to 60 days in soil B and 90 days in soil A.

## Discussion

Contamination of the environment with *M*. *bovis* is considered as essential for the persistence of bovine tuberculosis in animals and its interspecific indirect transmission. Although the survival of this mycobacteria in soil has been documented [13–15, 19], limited information is available on the soils used and on the edaphic factors that may affect *M*. *bovis* persistence. These experiments have dealt with controlled or environmental parameters that promote or hinder *M*. *bovis* persistence in soil such as temperature, moisture or UV exposition, but to our knowledge, impact of the soil type has not been thoroughly addressed. This study was designed to evaluate temperature and soil type impact on *M*. *bovis* survival.

Our overall results showed that *M*. *bovis* “Côte d’Or” may survive several months in soil under different laboratory conditions in two contrasted soils. Moreover, it was observed that low temperature (i.e. 4°C) strongly promotes *M*. *bovis* persistence whatever the soil type while warmer temperature (i.e. 22°C) shortens its survival time. Our results contrast with those of a previous laboratory study on *M*. *bovis* persistence [14], which showed that mycobacterial survival was longer at 25°C than at 4°C in a sterile sandy soil (pH 6.5–6.8, 60 days *vs* 16 days). However, our findings are in agreement with previous field studies which demonstrated that lower temperature increased *M*. *bovis* survival time up to 88 days [15, 19]. Moreover, we have demonstrated that soil type may impact the length of *M*. *bovis* survival. Although *M*. *bovis* survival was not significantly modified by soil characteristics when soils were incubated at 22°C, soil A that exhibits high clay and loam content and slightly basic pH allowed a one month longer survival of cultivable *M*. *bovis* (90 days *vs* 60 days) than the acidic sandy loamy soil B. Conclusions were hampered at 22°C since CFU numbers reached the detection limit of the culture method after 60 days of incubation. On the opposite, at 4°C, a significant difference in *M*. *bovis* survival was observed between the two soils, with a better survival in soil A than in soil B, suggesting an impact of soil type on *M*. *bovis* survival at lower temperatures. Enrichment broth could be very useful to recover low number of bacteria in complex matrices, such as *Salmonella typhimurium* in food or *Listeria monocytogenes* in soil [29, 30]. Such enrichments cannot be used to improve the detection of *M*. *bovis* since no mycobacterial selective broth is available to enrich mycobacteria from complex matrices like soils.

The clay soil with slightly basic pH seemed to promote *M*. *bovis* “Côte d’Or” survival which disagrees with previous studies suggesting that *M*. *avium* ssp. *paratuberculosis* and *M*. *bovis* survived better in sandy soils with acidic pH [21, 24, 26, 31]. However, Ghodbane *et al*. [16] demonstrated a one year persistence of cultivable *M*. *bovis* in a sandy soil with a basic pH (8.3) spiked with 10^8^ CFU g^- 1^, suggesting that *M*. *bovis* may survive a long time in soils harbouring a large range of pH. Finally, since the iron content is significantly higher in soils A and B from our study than the one in soils described in previous studies, this parameter may also be taken into account to explain the various results obtained for the survival of *M*. *bovis*. Further work should address this question by inoculating *M*. *bovis* to a large panel of contrasted soils and using a correlation statistical approach to decipher which are the major soils parameters affecting *M*. *bovis* survival length.

On the other hand, *M*. *bovis* DNA was detected by qPCR over a longer period of time than CFU (cultural approach) in soil samples incubated at 22°C, whatever the soil type. As a previous study reported that DNA of dead *M*. *bovis* cells was no longer amplified 10 days after cell death [14], we firstly hypothesized that our qPCR assay detected living *M*. *bovis* up to 150 days after inoculation of soils. Viable *M*. *bovis* cells have already been detected in sterile and biotic soil over a 15 month period using a Reverse Transcription PCR (RT-PCR) assay targeting the 16S rRNA encoding gene [14]. Physiological state of pathogenic mycobacteria such as *M*. *bovis* in soil is unknown but dormancy or a viable but non cultivable state (VBNC) have been suggested to explain their survival in environmental substrates [32, 33]. These hypotheses are supported by the presence of dormancy-related genes in *M*. *bovis* genome [34]. However, a second hypothesis is that qPCR amplified nude DNA or DNA from *M*. *bovis* dead cells. These hypothesis could be tested by RNA detection with RT-qPCR or with photoreactive DNA-binding dye such as propidium monoazide (PMA). Further researches are thus needed to ascertain the long bacterial survival in soil and to understand the genetic mechanisms involved in soil persistence of *M*. *bovis*.

Our laboratory conditions had probably been far different from field conditions where temperature fluctuations, moisture variations, UV radiation and competition with abundant and diverse microorganisms are detrimental to mycobacteria survival. *M*. *bovis* survival length observed in this study is probably longer than survival length under environmental conditions. In order to tackle the gap of knowledge about parameters conditioning *M*. *bovis* survival in soils, laboratory studies have to be set up to determine major factors involved in *M*. *bovis* survival.

Significant differences in the detection of *M*. *bovis* DNA between biotic and sterile soils were observed with the qPCR-based assay since the first day of the experiment. This might reflect a qPCR bias: i.e., dilution of the target *M*. *bovis* DNA in variable concentrations of soil metagenomic DNA following soil DNA extraction from biotic or sterile soil samples.

Extensive studies on a larger number of well-characterised and contrasted soils are required to target the key edaphic factors that promote *M*. *bovis* survival in soil. Further work is needed to determine the physiological state and virulence of *M*. *bovis* soil populations detected by qPCR and to confirm the occurrence non-cultivable or dormant cells capable of resuscitation under specific growth conditions.

## Supporting information

S1 Table Results of enumeration of *M*. *bovis* from sterilized soil microcosms and qPCR detection of *M*. *bovis* in both sterilized and fresh soil microcosms at two temperatures. (DOCX)
