# Supplementary figures and images for: Impact of temperature and soil type on Mycobacterium bovis survival in the environment
Source: PLoS One. 2017 Apr 27;12(4):e0176315. doi: 10.1371/journal.pone.0176315 (PMC5407823; doi:10.1371/journal.pone.0176315)

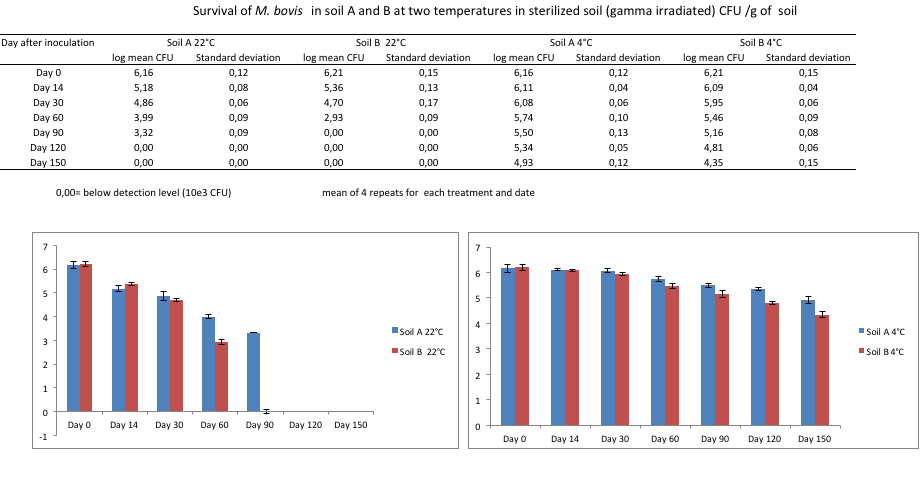


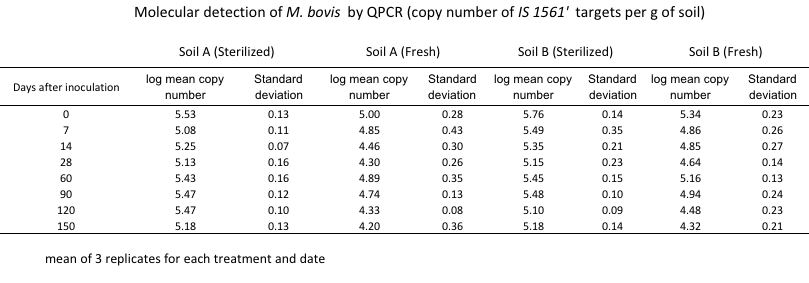

Supplement: S1 Table — (DOCX) [file pone.0176315.s001.docx]
